# Functional extracellular vesicles from SHEDs combined with gelatin methacryloyl promote the odontogenic differentiation of DPSCs for pulp regeneration

**DOI:** 10.1186/s12951-024-02542-0

**Published:** 2024-05-17

**Authors:** Hui Lu, Qing Mu, Weili Ku, Yexin Zheng, Ping Yi, Ling Lin, Pei Li, Boqun Wang, Jie Wu, Dongsheng Yu, Wei Zhao

**Affiliations:** Guanghua School of Stomatology, Hospital of Stomatology, Guangdong Provincial Key Laboratory of Stomatology, Sun Yat-sen University, Guangzhou, 510055 China

**Keywords:** Extracellular vesicles, Stem cells from human exfoliated deciduous teeth, Hydrogel, Odontogenic differentiation, Pulp regeneration

## Abstract

**Background:**

Pulp regeneration is a novel approach for the treatment of immature permanent teeth with pulp necrosis. This technique includes the combination of stem cells, scaffolds, and growth factors. Recently, stem cell-derived extracellular vesicles (EVs) have emerged as a new methodology for pulp regeneration. Emerging evidence has proven that preconditioning is an effective scheme to modify EVs for better therapeutic potency. Meanwhile, proper scaffolding is of great significance to protect EVs from rapid clearance and destruction. This investigation aims to fabricate an injectable hydrogel loaded with EVs from pre-differentiated stem cells from human exfoliated deciduous teeth (SHEDs) and examine their effects on pulp regeneration.

**Results:**

We successfully employed the odontogenic induction medium (OM) of SHEDs to generate functional EV (OM-EV). The OM-EV at a concentration of 20 µg/mL was demonstrated to promote the proliferation and migration of dental pulp stem cells (DPSCs). The results revealed that OM-EV has a better potential to promote odontogenic differentiation of DPSCs than common EVs (CM-EV) in vitro through Alizarin red phalloidin, alkaline phosphatase staining, and assessment of the expression of odontogenic-related markers. High-throughput sequencing suggests that the superior effects of OM-EV may be attributed to activation of the AMPK/mTOR pathway. Simultaneously, we prepared a photocrosslinkable gelatin methacryloyl (GelMA) to construct an OM-EV-encapsulated hydrogel. The hydrogel exhibited sustained release of OM-EV and good biocompatibility for DPSCs. The released OM-EV from the hydrogel could be internalized by DPSCs, thereby enhancing their survival and migration. In tooth root slices that were subcutaneously transplanted in nude mice, the OM-EV-encapsulated hydrogel was found to facilitate dentinogenesis. After 8 weeks, there was more formation of mineralized tissue, as well as higher levels of dentin sialophosphoprotein (DSPP) and dentin matrix protein-1 (DMP-1).

**Conclusions:**

The effects of EV can be substantially enhanced by preconditioning of SHEDs. The functional EVs from SHEDs combined with GelMA are capable of effectively promoting dentinogenesis through upregulating the odontogenic differentiation of DPSCs, which provides a promising therapeutic approach for pulp regeneration.

**Supplementary Information:**

The online version contains supplementary material available at 10.1186/s12951-024-02542-0.

## Background

Pulp necrosis arising from caries, dental trauma, and dental deformities seriously compromises the long-term prognosis of immature permanent teeth. Instead of traditional root canal treatment, pulp regeneration is considered as a promising alternative to restore the physiological function of dental pulp [1]. The regenerative engineering approach involves the combination of stem cells, scaffolds, and growth factors. Up till now, some attempts have been made to implant stem cells from human exfoliated deciduous teeth (SHEDs) in the root canals of young permanent teeth to regenerate dental pulp tissue and promote continuous root growth [2].

In recent years, extracellular vesicles (EVs) secreted by mesenchymal stem cells (MSCs) have become the focus of attention in the field of tissue regenerative therapies due to their low immunogenicity and higher safety [3]. Since EVs carry cell-type specific biological “cargo” containing proteins, lipids, and RNA to participate in cell-to-cell communication, EVs secreted by MSCs may have therapeutic effects similar to those of their parental cells [4]. There is also evidence that EVs derived from dental pulp stem cells (DPSCs) are capable of enhancing angiogenesis and osteogenesis [5–7], offering that EVs derived from DPSCs may be a biomimetic tool to improve pulp regeneration in teeth with pulp necrosis. Given that SHEDs share convenient accessibility and higher osteogenic potential compared to DPSCs, EVs derived from SHEDs have been extensively investigated in the field of bone regeneration [5, 8, 9]. Recently, our team proved that EVs derived from SHEDs have superior osteogenic effects to those derived from DPSCs [10]. However, the role of SHED-derived EVs in promoting dentinogenesis has not been clearly realized yet.

The physiological state of cells also affects the function of EVs. External stimulation and pre-culture are some of the engineering strategies to tailor EVs for better therapeutic potency in tissue regeneration [11]. By altering the cell culture medium or inducing cells to differentiate in a specific direction, the EVs secreted by their parental cells will possess corresponding biological effects [11, 12]. Although a better osteogenic/odontogenic capacity of EVs than pre-differentiated cells was demonstrated in the literature [13–15], comprehensive evaluations of preconditioned EVs on the biological behavior of recipient cells remain limited. To choose EVs with better therapeutic potency, this investigation essentially focuses on characterizing the influences of SHED-derived EVs at various concentrations on cell proliferation and migration, especially odontogenic potential, in an attempt to prepare an ideal “messenger” for pulp regeneration.

Generally, dental pulp regeneration requires a suitable scaffold to provide three-dimensional (3D) spatial location and regulate stem cell differentiation, proliferation, or metabolism. Further, suitable scaffold materials are able to effectively protect EVs from rapid clearance and degradation [16]. Hydrogels represent a class of materials with a hydrophilic 3D polymer network that can be prepared by natural materials such as gelatin or synthetic polymers such as polyethylene glycol through chemical cross-linking, photopolymerization, thermoplastic, or freezing. With good elasticity, biocompatibility, tunable degradability, and mechanical properties, they have been broadly employed to simulate biological delivery [17]. Gelatin methacrylate (GelMA) combines the properties of natural and synthetic biomaterials and is extensively utilized for tissue engineering due to its appropriate injectability, thermosensitivity, and photo-cross-linkability [18–20]. The GelMA hydrogel can be prepared in an injectable form, allowing it to be injected into the pulp cavity and root canal before gelling. This feature makes it a promising material for exploitation in dental pulp regeneration research; nevertheless, limited research works have been devoted to the use of MSCs-derived EVs combined with hydrogel for dental pulp regeneration, and the biological properties of EV-encapsulated hydrogels have not been fully examined yet.

In the present investigation, we isolate functional EVs from pre-differentiated SHEDs (OM-EV) and compare their effects with common EVs (CM-EV). We then employ GelMA to encapsulate OM-EV and proceed with developing a biocompatible hydrogel complex to upregulate the odontogenic differentiation of DPSCs in vivo. To this end, we hypothesize that GelMA hydrogel is capable of sustaining the release of OM-EV and promoting the odontogenic differentiation of DPSCs for dentinogenesis. To the best of our knowledge, this is the first investigation to combine SHED-engineered EV with hydrogel for pulp regeneration, which may provide a promising biomimetic tool in pulp regeneration.

## Methods

### Culture and characterization of human SHEDs and DPSCs

Caries-free exfoliated deciduous teeth were collected from children aged 6 to 10 years at the Department of Pediatric Dentistry, Hospital of Stomatology, Sun Yat-sen University. The required informed consent was obtained from individuals and their parents. Dental pulp was appropriately harvested from extracted teeth under aseptic conditions and then digested to isolate SHEDs as elucidated in Refs [21, 22]. The SHEDs were cultured in high-glucose Dulbecco’s modified Eagle medium (DMEM, Gibco, Grand Island, USA) supplemented with 10% fetal bovine serum (FBS, Gibco) and 1% penicillin/streptomycin (P/S; Gibco, USA) at 37 °C. Cells at passages 3–5 (P3–P5) were adopted for subsequent experiments. The DPSCs were obtained from impacted third molars of 18-25-year-old patients at the Department of Oral and Maxillofacial Surgery with the same protocol.

The cell morphologies of SHEDs and DPSCs at P0 and P3 were observed by an inverted microscope (Zeiss Axio Observer, Oberkochen, Germany). The colony formation method was utilized to identify the proliferation ability of SHEDs and DPSCs. To examine MSCs-specific markers, cells were suitably stained with antibodies (CD34, CD44, CD45, CD73, CD90, and CD105, Biolegend, China) and analyzed by flow cytometry (Beckman Coulter, USA). To determine the multipotentiality of SHEDs and DPSCs, cells were treated with an osteogenic induction medium consisting of α-MEM, 10% FBS, 1% P/S, 50 µmol/L ascorbic acid (Sigma-Aldrich), 10 mmol/L β-glycerophosphate (Sigma-Aldrich), and 100 nmol/L dexamethasone (Sigma-Aldrich) for 14 days, and then stained with Alizarin red solution (Cyagen Biosciences Inc., China). An adipogenic induction medium (Cyagen Biosciences, China) was implemented to induce adipogenic differentiation of DPSCs for 21 days, and Oil red O staining was applied to assess lipid droplet formation.

### Isolation and purification of EVs

The SHEDs cell culture supernatants at P3-P5 were collected for EV isolation. For OM-EV isolation, SHEDs were cultured in the odontogenic induction medium (OM, α-MEM supplemented with 10% FBS, 1% P/S, 10 mmol/L β-glycerophosphate, 50 µmol/L ascorbic acid, and 100 nmol/L dexamethasone) for 10 days, and then were appropriately cultured in serum-free OM for 48 h. Serum-free conventional medium (CM, α-MEM with 1% P/S) of SHEDs was collected for CM-EV isolation, as described in some detail in Ref [15].

According to Refs [10, 23], ultracentrifugation for OM-EV and CM-EV isolation (under the conditions of 300 g for 10 min, 2000 g for 15 min, and 10,000 g for 30 min) to eliminate cell debris and large vesicles were utilized and then ultracentrifuged at 100,000 g for 90 min with an ultracentrifuge (Beckman Coulter Optima L-100XP, USA). To eliminate the influence of various cell-conditioned media, the pelleted EVs were washed twice with pre-cool PBS and centrifuged at 100,000 g for 90 min again. The cold PBS was employed to resuspend the deposits. The CM-EV and OM-EV protein concentrations were quantified with the microBCA Protein Assay Kit (CWbio, Shanghai, China).

Besides, EVs-depleted FBS was obtained by centrifugation at 100,000 g for 16 h with the ultracentrifuge (Beckman Coulter, USA) to deplete bovine EVs [24]. The EVs-depleted FBS was then employed in vitro experiments.

### Identification and characterization of CM-EV and OM-EV

The appropriate identifications of OM-EV and CM-EV were performed as suggested by the International Society for Extracellular Vesicles (ISEV) [25]. The morphology of EVs was observed through TEM (Hitachi H-7650, Japan) and AFM (Bruker, Karlsruhe, Germany). For atomic force microscopy (AFM), EVs in PBS were incubated on clean glass slides. The MLCT-F cantilevers with a nominal tip radius of 20 nm and a spring constant of 0.6 N/m were implemented for the required measurements. The dimensions and particle concentrations of EVs were determined by nanoparticle tracking analysis (NTA) using a ZetaView nanoparticle tracer (Particle Metrix, Germany). The EV markers, CD63 and TSG 101 (Zen-bio, China), were determined by Western blotting to identify the purity of EVs.

### EV labeling and uptake assay

To examine the incorporation of EVs, CM-EV, and OM-EV were labeled with Dil (Zetalife, USA) according to the manufacturer’s instructions. Briefly, PBS-suspended CM-EV and OM-EV were suitably stained with Dil (3 µL Dil in 1 mL DMSO) for 20 min at 4 ℃. The Dil-labeled EVs were then collected by ultracentrifugation at 100,000 g for 90 min. After that, DPSCs (10^4^ cells/dish) were appropriately seeded on the dishes. As cells reached 50% confluence, they were treated with Dil-labeled OM-EV or CM-EV (100 µg/mL). A negative control was established by adding an equal volume of PBS. Since Dil precipitates and Dil-labeled EVs may have similar size and fluorescence intensity, a Dil-only control was also applied (PBS negative control stained with Dil) to rule out the illusion caused by the lipophilic dye [26]. After coculturing for 2, 6, 12, 24, 48, 72, 96, 120, and 168 h, the cells were fixed with paraformaldehyde, and stained with phalloidin (Solarbio, China) and 6-diamidino-2-phenylindole (DAPI) (Beyotime, Shanghai, China). The internalization of CM-EV and OM-EV by DPSCs was then suitably visualized with a confocal laser scanning microscope (CLSM, FV3000, Olympus, Japan).

### Assessment of the proliferation ability

To evaluate the effect of CM-EV and OM-EV on the proliferation of DPCSs, the CCK-8 Kit (Dojindo, Kumamoto, Japan) was utilized. For this purpose, DPSCs were seeded in 96-well plates (5000 cells/well) and cultured with conditioned media containing various concentrations of CM-EV or OM-EV (0, 1, 5, 10, and 20 µg/mL, respectively). After 1, 3, 5, and 7 days, 10% CCK-8 solution was added to each well. After incubating the plates at 37 °C for 1 h, the optical density (OD) value at 450 nm was appropriately measured with a microplate reader (Tecan, Infinite f200 PRO, Switzerland). To further confirm the proliferative effects of CM-EV and OM-EV, the EdU Proliferation Kit (Beyotime, China) was utilized. The DPSCs were seeded in dishes at a density of 10^4^ cells per well and then incubated with 20 µg/mL CM-EV and OM-EV for 7 days. After staining the cell nuclei with Adize488 and DAPI, six random fields from each group were taken by a laser confocal microscope (Zeiss LSM 980, Germany). The proportion of Edu-positive nuclei in each group was calculated with Image J (National Institutes of Health, Bethesda, MD). Additionally, the cell cycle profiles after 20 µg/mL CM-EV and OM-EV treatment were established by flow cytometry.

### Assessment of the migration ability

The migration ability of DPSCs was evaluated using transwell chambers with 12 μm pores (Corning, USA). A volume of 100 µL suspension containing 10^5^ DPSCs in FBS-free α-MEM was seeded in the upper chamber. In the lower chamber, 500 µL α-MEM containing 10% EV-depleted FBS and 20 µg/mL CM-EV or OM-EV was added. After 24 h, the migrated DPSCs on the lower surface of the membrane were appropriately fixed with paraformaldehyde, stained with 0.1% crystal violet, and recorded with an inverted microscope.

### Assessment of the odontogenic potential in vitro

To evaluate the effect of CM-EV and OM-EV on the formation of calcium nodules, DPSCs were seeded in 12-well plates with a density of 1 × 10^5^ cells per well and treated with an odontogenic medium containing 20 µg/mL CM-EV. or OM-EV. After 7 and 14 days, cells were fixed with 4% paraformaldehyde and stained with Alizarin red solution to visualize calcium nodules under an inverted microscope. Then, the calcium nodules were measured by incubating with cetylpyridinium chloride, and OD values were measured at 562 nm wavelength. In addition, alkaline phosphatase (ALP) activity assay and staining were performed after 7 days of odontogenic induction with an ALP assay kit (Nanjing Jiancheng Bioengineering Institute, China) and a NBT/BCIP staining kit (Beyotime, China), following the manufacturer’s instructions.

### Western blot analysis

The protein expression of odontogenic-related genes, including dentin sialophosphoprotein (DSPP), dentin matrix acidic phosphoprotein 1 (DMP-1), alkaline phosphatase (ALP), and runt-related transcription factor 2 (Runx2), was detected by Western blot analysis. The DPSCs were appropriately cultured in an odontogenic medium containing either 20 µg/mL CM-EV or OM-EV for 7 and 14 days as explained above. Cells were then collected and lysed in RIPA buffer (KeyGen BioTECH, Nanjing, China) containing a 1% protease inhibitor cocktail (CWBIO, China). The protein concentration was determined by employing the BCA assay kit (CWBIO). The proteins of each group were separated by 10% SDS-PAGE (GenSpirt, China) and transferred to PVDF membranes (Millipore, USA). After blocking with 5% fat-free milk, the PVDF membranes were subsequently incubated overnight at 4 °C with primary antibodies anti-DSPP (1:500, Novus, USA), anti-DMP-1 (1: 500, Bioss), anti-Runx2 (1:1000, Novus), anti-ALP (1:1000, Novus), anti-GAPDH (1:1500, Novus), followed by incubation with secondary antibody (1:5000, GNI, Japan). An advanced chemiluminescence detection system (Millipore) was utilized to detect immunoblots with an imaging system (ChemiDoc, Bio-Rad, China). The bands’ intensities were suitably measured via ImageJ and normalized to GAPDH.

### qRT-PCR

The mRNA expressions of DSPP, DMP-1, ALP, and Runx2 were appropriately detected via qRT-PCR. The total RNA was extracted from DPSCs treated with odontogenic medium supplemented with 20 µg/mL OM-EV and CM-EV for 7 and 14 days, using the RNA-Quick Purification Kit (YiShan Biotech, China). The RNA’s concentration and purity were suitably determined on an ND-1000 spectrophotometer (NanoDrop Technologies, Santa Clara, CA). The cDNA was synthesized with PrimeScript RT Master Mix (Takara, Japan). The qRT-PCR was performed via the SYBR-green PCR kit (Yeasen Biotechnology, China) on a LightCycler 96 system (Roche, Basel, Switzerland). The relative mRNA expression levels of the target genes were normalized to GAPDH. The primer sequences were provided in Supplementary Table 1.

### MicroRNA sequencing

Since EVs carry microRNAs to modulate post-transcriptional gene expression in recipient cells, we applied microRNA sequencing to explore the mechanisms underlying the biological function of EVs. Small RNAs were extracted from CM-EV and OM-EV (*n* = 3 for each) using an Exosome RNA Purification Kit (EZBioscience, USA) following the manufacturer’s instructions. MicroRNA libraries were constructed and subjected to deep sequencing via the Illumina Hi-Seq 2500 platform at RiboBio Co. Ltd. (Guangzhou, China). Differentially expressed miRNAs with a 1.3-fold change in expression (*P* < 0.05) were analyzed. Kyoto Encyclopedia of Genes (KEGG) pathway analysis. Additionally, gene ontology (GO) analysis of target mRNA genes of differentially expressed miRNAs was performed to explore signaling pathways potentially involved in OM-EV function. To confirm whether the AMPK/mTOR pathway is involved in the regulation of OM-EV-mediated odontogenic differentiation, Western blotting was conducted to assess the expression of p-AMPK and p-mTOR in DPSCs after incubation with OM-EV for 7 days. The antibodies used were as follows: anti-AMPK (1:1000, Affinity, USA), anti-p-AMPK (1:800, Affinity, USA), anti-mTOR (1:1000, Affinity, USA), and anti-p-mTOR (1:800, Affinity, USA).

### Construction and characterization of OM-EV-encapsulated hydrogel

To construct an OM-EV-encapsulated hydrogel, the gelatin methacryloyl (GelMA, Engineering for Life, China) was prepared according to the manufacturer’s instructions. The effects of GelMA on the vitality and morphology of DPSCs were tested by co-culturing DPSCs with various concentrations of GelMA (5, 15, and 30%). The DPSCs/GelMA complex was crosslinked with a 405 nm visible light source for 30 s. The CCK-8 kit, EdU proliferation kit, and immunofluorescence staining for the cytoskeleton were applied to assess cell viability and morphology in GelMA.

After determining the optimal hydrogel concentration, OM-EV in PBS was gently mixed with 5% GelMA and UV-crosslinked. The release efficiency of OM-EV from the hydrogel was then measured via the microBCA protein assay kit. Briefly, 100 µL of OM-EV-encapsulated hydrogel was placed in a 48-well plate and incubated with 700 µL of PBS at 37 °C. The concentration of OM-EV released from the hydrogel was determined on days 1, 3, 5, and 7. Since we expected that the minimum effective concentration of OM-EV would be able to sustain for at least 7 days, the OM-EV encapsulated in GelMA was determined by multiplying the amount of OM-EV released on day 7 by 20 µg/mL. After that, an OM-EV-encapsulated hydrogel was constructed by embedding OM-EV (500 µg/mL) in 5% GelMA. The microstructure of the OM-EV-encapsulated hydrogel was then observed by SEM (Philips XL30 FEG microscope, Eindhoven, The Netherlands) after lyophilization.

### Uptake and biocompatibility experiments with OM-EV-encapsulated hydrogel in vitro

To observe the distribution of DPSCs and OM-EV in the hydrogel, DPSCs were mixed at a density of 10^7^/mL with OM-EV-encapsulated hydrogel, where OM-EV was pre-labeled with Dil. After 72 h of culture, the CLSM was utilized to visualize the distribution of phalloidin-labeled DPSCs and Dil-labeled OM-EVs. The internalization of OM-EV released from the hydrogel by DPSCs was also captured with CLSM.

The viability of DPSCs in OM-EV-encapsulated hydrogel was detected using a live/dead cell staining kit (Procell, China) according to the manufacturer’s protocol. After 3 and 7 days of cultivation, the number of dead cells (stained red) was examined by CLSM and measured by ImageJ from eight random fields.

To evaluate the effects of OM-EV-encapsulated hydrogel on the migration ability of DPSCs, two additional assays were performed. The migration distance of DPSCs in the hydrogel was recorded with an inverted microscope on days 3 and 7. Additionally, a transwell assay was applied to detect whether the hydrogel had a “cell homing” effect on DPSCs. After one day of co-culture, the cells that migrated to the transwell chamber’s lower surface were stained with 0.1% crystal violet and recorded.

### Subcutaneous transplantation in the tooth root slice model

Subcutaneous transplantation with a root slice model was performed in nude mice to examine the effects of hydrogel encapsulated with OM-EV on dentin formation in vivo. Six-week-old BALB/c-nu mice were obtained from the Laboratory Animal Center of Sun Yat-sen University. Experimental procedures were approved by the Institutional Animal Care and Use Committee of Sun Yat-Sen University (No. SYSU-IACUC-2023-001497). The nude mice were randomly divided into four groups of three: “hydrogel + PBS”, “hydrogel + DPSCs”, “hydrogel + DPSCs + CM-EV”, and “hydrogel + DPSCs + OM-EV”.

To prepare the tooth root slice, extracted human premolars were collected and the root canal space was prepared with an inner diameter of 2 mm and a height of 3 mm. Before transplantation, DPSCs (10^7^/mL) were mixed with GelMA and corresponding EVs (500 µg/mL). A volume of 50 µL of EV-encapsulated hydrogel embedded with DPSCs was injected into the root canal spaces. After being cultured for one day in vitro, tooth slices were subcutaneously transplanted on the back of nude mice under abdominal anesthesia with 2% sodium pentobarbital (as demonstrated in Fig. 7A). The samples were harvested 8 weeks after transplantation.

### Histological and immunohistochemistry analysis

After sample collection, the dental root slices were fixed in 10% buffered formalin for 24 h. Following decalcification with 10% EDTA and embedding in paraffin, 6 μm-thick slices were prepared. For histological analysis, the sections were stained with hematoxylin and eosin (H&E) and Masson’s trichrome staining solution (Beyotime, China). The tissue formation in the root canal of the tooth slices was observed under the light microscope. Immunohistochemical (IHC) staining was performed via the Histostain-SP (streptavidin-peroxidase) kit (Bioss, China) based on the manufacturer’s protocol. The tissue sections were deparaffinized, heat retrieved, blocked, and then incubated with primary antibodies: anti-DSPP (1:200, Novus, USA) and anti-DMP1 antibody (1:200, Bioss, China) at 4 °C overnight, followed by incubation with HRP-conjugated and DAB staining to show signals. For quantitative immunohistochemical analysis, the percentages of DSPP- and DMP-1-positive cells were calculated by dividing the number of brown-stained cells by the total number of cells in the observation area (*n* = 3).

### Statistical analysis

SPSS 20.0 software (SPSS Inc., Chicago, USA) was utilized to conduct statistical analysis. Student’s t-test or one-way analysis of variance (ANOVA) was appropriately implemented to identify significant discrepancies among different groups, and a *P*-value < 0.05 was considered statistically significant.

## Results

### Characterization of SHEDs and DPSCs

Human SHEDs and DPSCs were successfully cultured and characterized (Fig. S1). SHEDs and DPSCs both exhibited a spindle-like or fibroblast-like morphology, with SHEDs generally slightly smaller in size (Fig. S1A). Colony formation assay demonstrated that both cell types were capable of forming colonies, but the proliferative ability of SHEDs was stronger than that of DPSCs (Fig. S1B). After induction of osteogenesis and adipogenesis, calcium deposits and lipid droplets were observed (Fig. S1C). Flow cytometric analysis (Fig. S1D) revealed that both SHEDs and DPSCs expressed markers associated with MSCs (CD44, CD73, CD90, and CD105) and were negative for the expression of hematopoietic markers (CD34 and CD45). These results are indicative of the fact that cells isolated from human exfoliated deciduous teeth and impacted third molars are mesenchymal stem cells with active proliferation capacity and multiple differentiation potential.

### Identification of CM-EV and OM-EV

**Fig. 1:**
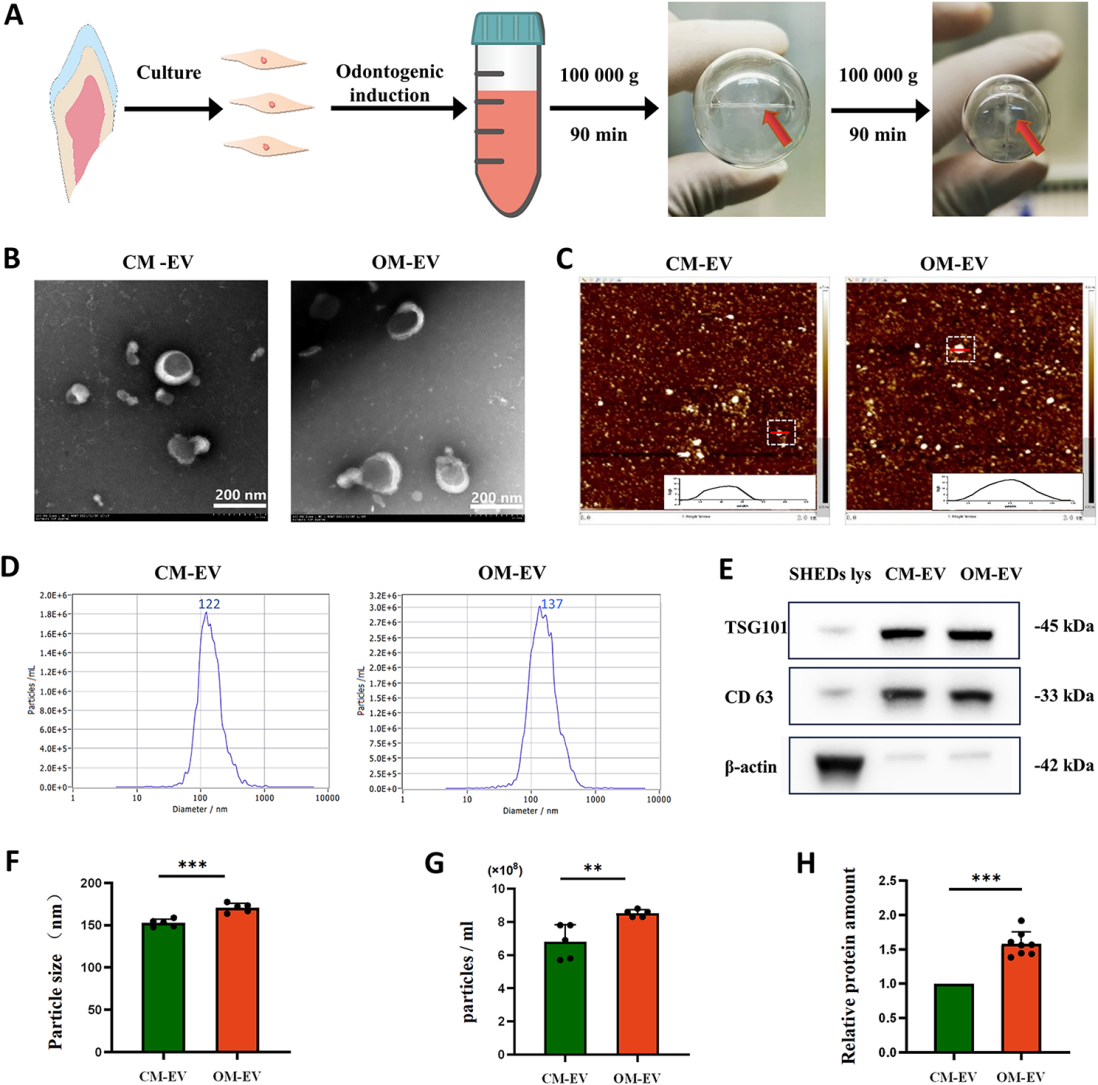
Isolation and identification of OM-EV and CM-EV: **A** Schematic representation showing the preparation and isolation of OM-EV and CM-EV. **B** Morphology of OM-EV and CM-EV under TEM (note: Scale bar represents 200 nm). **C** Saucer-like morphology of OM-EV and CM-EV under AFM. **D** The presented particle distributions of OM-EV and CM-EV by NTA. **E** Detection of EV surface markers of TSG101 and CD63 by Western Blotting. **F** Comparison of particle size. **G** Comparison of particle concentration (EVs from 9 mL supernatant were resuspended in 2.5 mL ddH_2_O). **H** Comparison of protein amount. (note: Error bars represent the values of “mean ± s.d.”; ** *P* < 0.01, *** *P* < 0.001)

The CM-EV and OM-EV were appropriately identified by TEM, AFM, NTA, and Western blotting. The taken TEM images showed the round morphology of CM-EV and OM-EV (Fig. 1B). The AFM images also demonstrated vesicles with a round morphology and a diameter between 60 and 80 nm (Fig. 1C). The results obtained from NTA showed that the diameters of CM-EV and OM-EV are placed in the interval of 50–200 nm (see Fig. 1D), with average values of 152 nm and 170 nm. The Western blot analysis also revealed that OM-EV and CM-EV were positive for CD63 and TSG101 and negative for β-actin (see Fig. 1E). These findings confirmed that OM-EV and CM-EV were successfully isolated and identified.

In addition, to evaluate the secretory efficiency of cells treated with CM and OM, the amounts of particles and protein of CM-EV and OM-EV were appropriately compared (see Fig. 1F, G and H). The obtained results revealed that OM-EV possessed larger sizes, higher particle concentrations, and greater protein amounts compared to CM-EV.

### Uptake of OM-EV by DPSCs

**Fig. 2:**
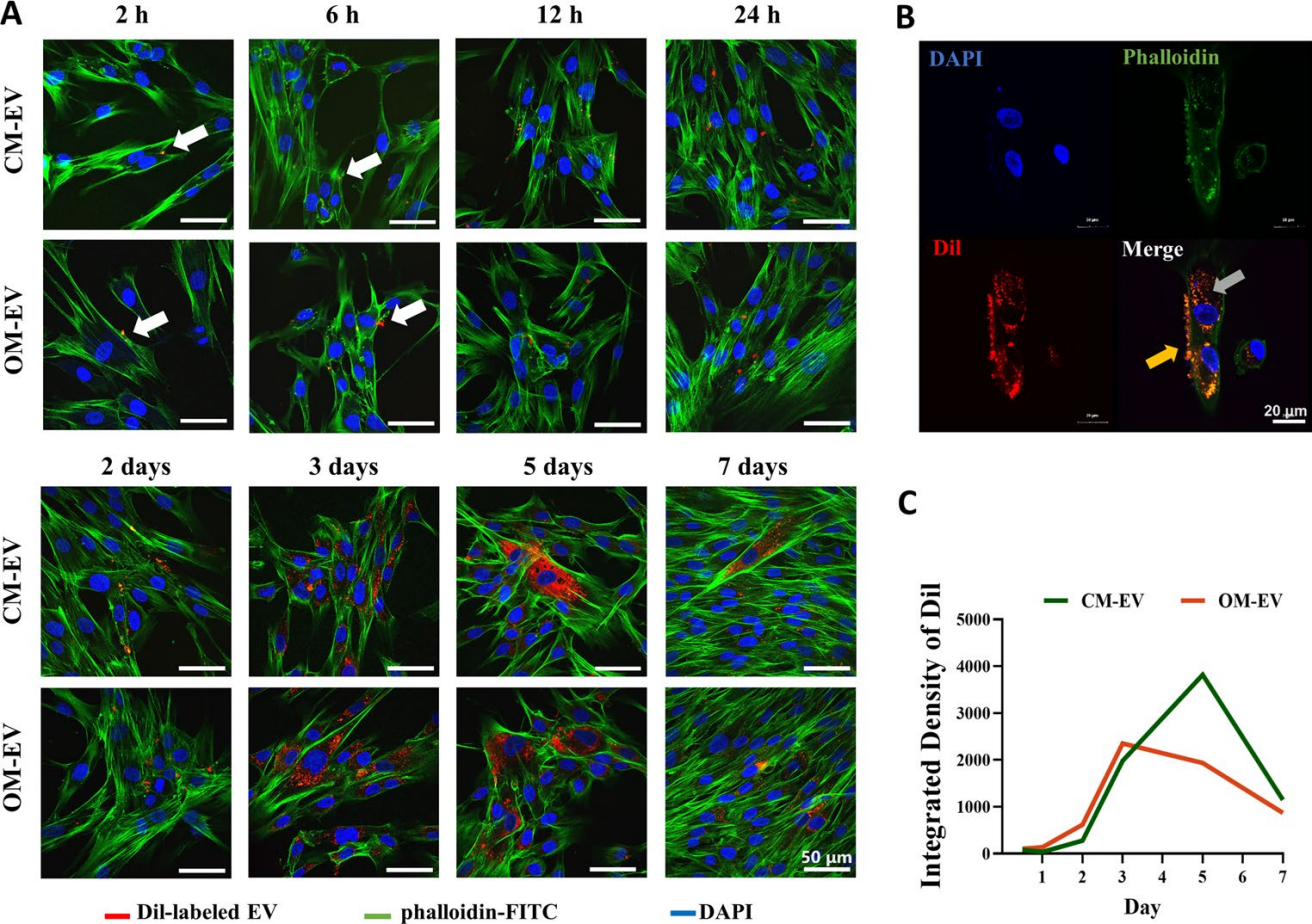
The uptake of OM-EV and CM-EV by DPSCs: **A** The internalization of Dil-labeled OM-EV and CM-EV (white arrow) by DPSCs observed in 7 days (note: Scale bar represents 50 μm). **B** A representative cross-section of the 3D reconstruction showing the localization of EVs inside (gray arrow) and outside (yellow arrow) the cell on day 3 (note: Scale bar represents 20 μm). **C** Integrated density of Dil in DPSCs treated with OM-EV and CM-EV in 7 days

The uptake of OM-EV by DPSCs was also confirmed with CLSM (see Fig. S2 and Fig. 2). Under the same laser intensity, the Dil-only control did not exhibit obvious red fluorescence (Fig. S2), indicating that the bright red fluorescence within DPSCs in Fig. 2A must be attributed to Dil-labeled EVs. As illustrated in Fig. 2, after 2 h of co-culture, only a few labeled EVs were attached to the cell membrane of DPSCs. However, after 2 days, a certain number of EVs entered the cells. After 3–5 days, the accumulation of EVs in the cells reached its peak. At day 7, endocytosed EVs were still seen inside the cells, although labeled EVs had been removed from the culture medium on day 3 (Fig. 2A). The EVs were primarily located in the cytoplasm around the nucleus, and a considerable amount of EVs was attached to the cell membrane (see Fig. 2B). A quantification analysis of the Dil integrated density revealed that the accumulation of OM-EV in DPSCs was slightly higher than that of CM-EV in the first 3 days, while more CM-EV could be observed in the following 4 days (see Fig. 2C).

### OM-EV mildly promoted the proliferation and obviously enhanced the migration of DPSCs

**Fig. 3:**
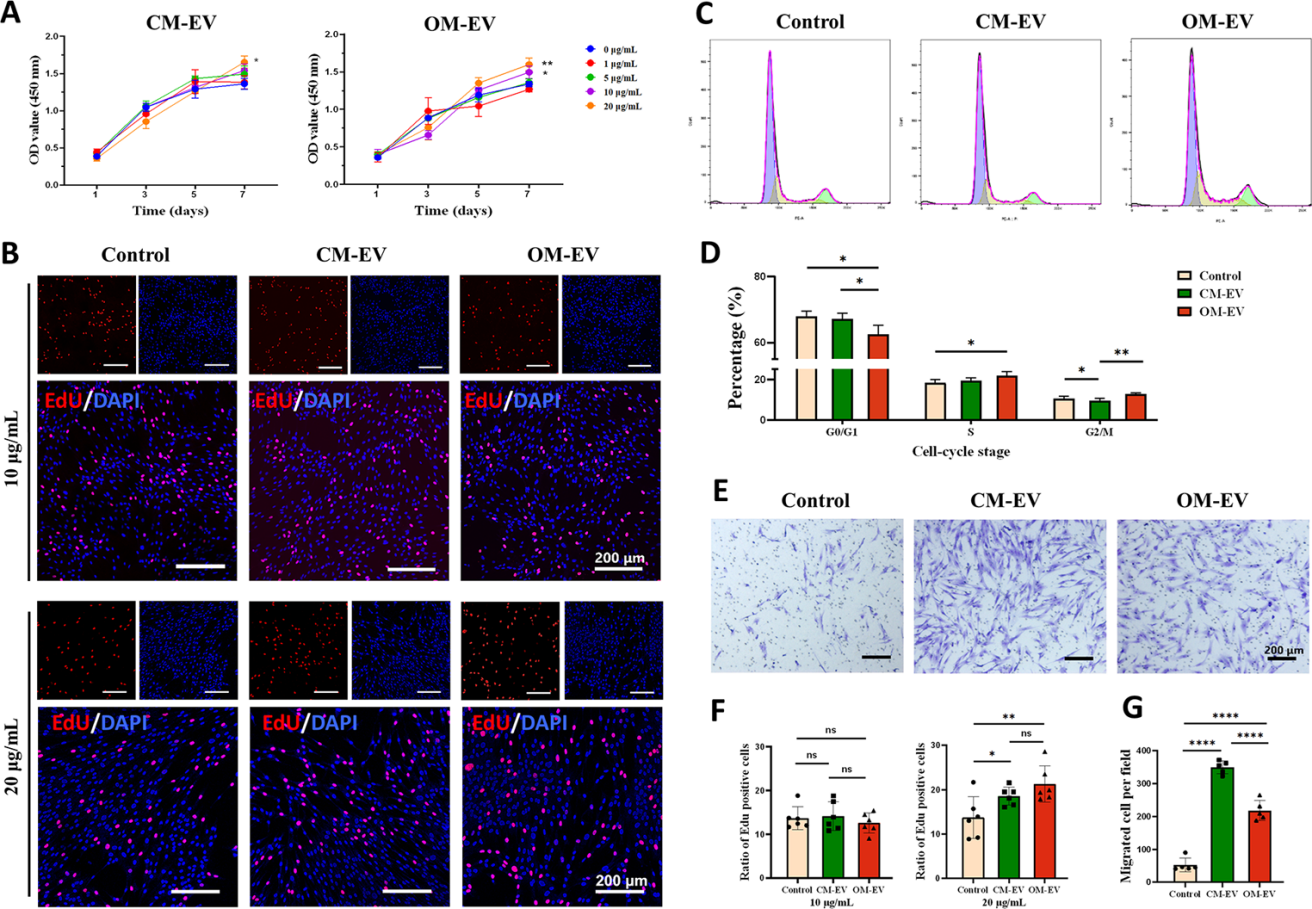
The proliferation and migration of DPSCs promoted by the uptake of OM-EV. **A** Proliferation of DPSCs cocultured with various concentrations of CM-EV or OM-EV for 7 days assessed by CCK-8 assay (* *P* < 0.05, ** *P* < 0.01 vs. the control group). **B** Effects of 10 and 20 µg/mL CM-EV and OM-EV on the proliferation of DPSCs detected by EdU proliferation assay (note: Scale bar represents 200 μm). **C** Effect of 20 µg/mL CM-EV or OM-EV on the cell cycle of DPSCs. **D** Distribution of cell cycle stage in each group. **E** Representative images showing the capacity of DPSC migration under the treatment of 20 µg/mL CM-EV or OM-EV tested by the transwell assay (note: Scale bar represents 200 μm). **F** The ratio of EdU-positive cells under the treatment of 10 and 20 µg/mL CM-EV and OM-EV. **G.** Comparison of migrated cells per field (note: Error bars represent the values of “mean ± s.d.”; * *P* < 0.05, ** *P* < 0.01, *** *P* < 0.001, and **** *P* < 0.0001)

The proliferation of DPSCs was found to be promoted by both CM-EV and OM-EV at a concentration of 20 µg/mL, as determined by the CCK-8 assay (see Fig. 3A). This proliferative effect was further verified by the EdU assay (see Fig. 3B and F). The treatment with 10 µg/mL CM-EV or OM-EV did not remarkably affect the proportion of EdU-positive cells compared to the control group. However, at a concentration of 20 µg/mL, the proportion of EdU-positive cells in both groups was clearly higher than that of the control group (18.03% vs. 13.74%, *P* < 0.05, and 21.28% vs. 13.74%, *P* < 0.01), but there was no statistically significant discrepancy between the CM-EV group and the OM-EV group (*P* > 0.05). Furthermore, the performed cell cycle analysis (see Fig. 3C and D) indicated that the percentage of S-phase cells increased from 18.8 to 20.9% after treatment with 20 µg/mL OM-EV for 3 days, while the proportion of G0/G1 phase cells decreased from 67.9 to 62.9% with a statistically significant difference of *P* < 0.05. Therefore, a concentration of 20 µg/mL was selected for subsequent experiments.

Transwell test results also revealed that the migration potency of DPSCs could be improved by CM-EV and OM-EV at a concentration of 20 µg/mL (*P* < 0.0001), with the CM-EV group performing slightly better than the OM-EV group (see Fig. 3E and H).

### OM-EV-based enhancement of the odontogenic potential of DPSCs

**Fig. 4:**
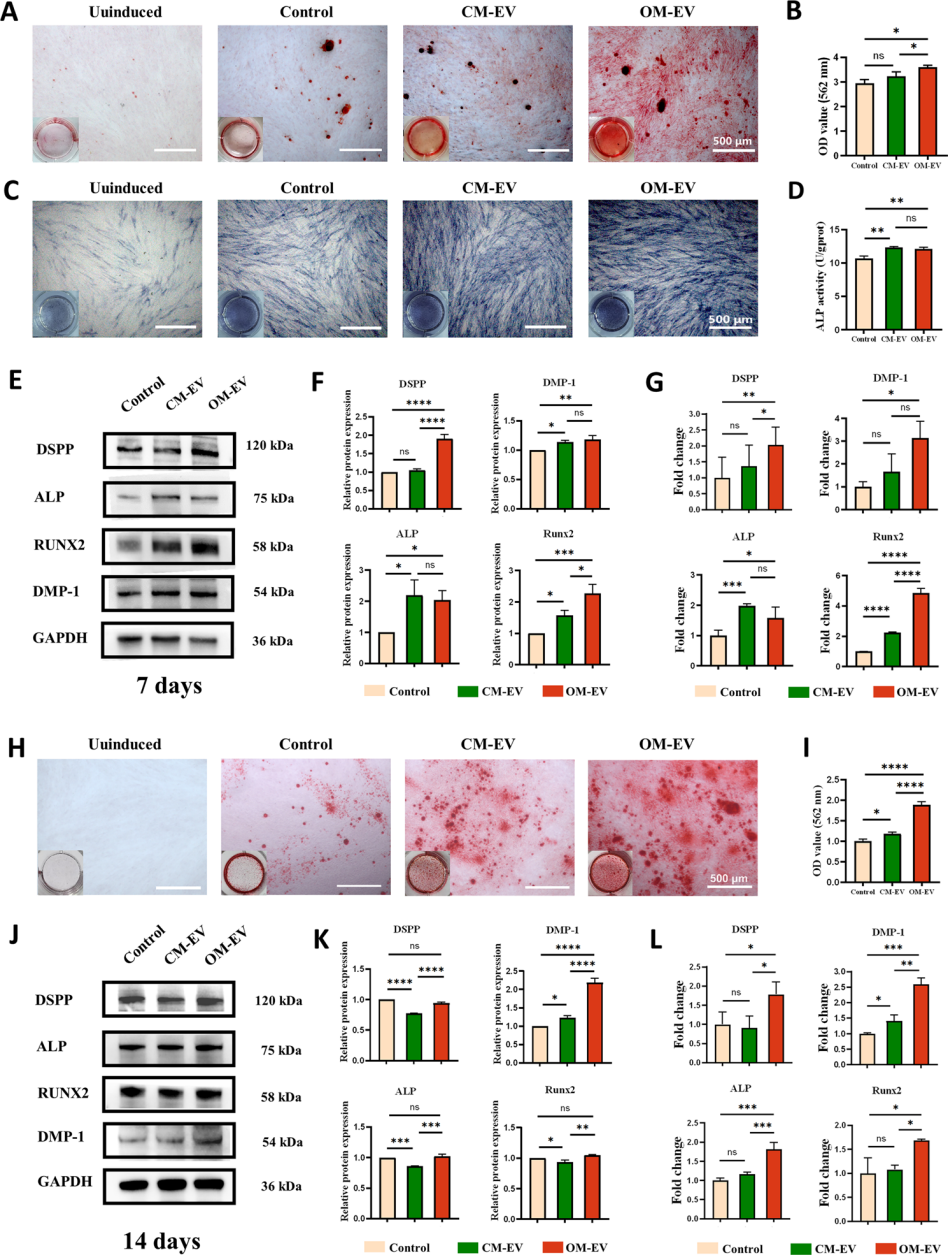
Assessment of odontogenic potential of DPSCs after culture with odontogenic induction medium containing OM-EV. **A** Gross appearance and microscopic images of Alizarin red staining for 7 days (note: Scale bar represents 200 μm). **B** Quantitation analysis of Alizarin red staining for 7 days. **C** Gross appearance and microscopic images of ALP staining for 7 days (note: Scale bar represents 200 μm). **D** Quantitative detection of ALP activities after 7 days. **E** Expression levels of odontogenic-related proteins for 7 days. **F** Quantification of the gray signal intensity based on the Western blotting at day 7. **G** Expression levels of odontogenic genes tested by qRT-PCR at day 7. **H** Gross appearance and microscopic images of Alizarin red staining for 14 days (note: Scale bar represents 200 μm). **I** Quantitation analysis of Alizarin red staining for 14 days. **J** Expression levels of odontogenic-related proteins for 14 days. **K** Quantification of the gray signal intensity based on the Western blotting at day 14. **L** Expression levels of odontogenic genes tested by qRT-PCR at day 14 (note: * *P* < 0.05, ** *P* < 0.01, *** *P* < 0.001, and **** *P* < 0.0001)

To evaluate whether OM-EV is able to enhance the odontogenic potential of DPSCs, Alizarin red staining, ALP staining, ALP activity assessment, Western blot, and qRT-PCR were applied. After co-culturing with 20 µg/mL OM-EV for 7 and 14 days, Alizarin red staining and quantitative analysis indicated that OM-EV promoted the deposition of calcium nodules compared with CM-EV and the control group, especially at day 14 (*P* < 0.0001) (see Fig. 4A, B and H, and 4I). After induction for 7 days, ALP staining (Fig. 4C) and ALP activity detection (Fig. 4D) identified that OM-EV was capable of improving the alkaline phosphatase activity of DPSCs compared with the control (*P* < 0.01), although the difference between CM-EV and OM-EV groups was not obvious.

The expression of odontogenic differentiation-related genes and proteins in DPSC cells was detected by Western blotting and qRT-PCR. After 7 days of odontogenic induction, the protein expression levels of DSPP, DMP-1, ALP, and Runx2 were significantly increased in DPSCs treated with CM-EV and OM-EV, and the expression of DSPP and Runx2 was significantly higher in OM-EV group compared with CM-EV group (see Fig. 4E and F). After 14 days, the protein expression of DSPP, DMP-1, ALP, and Runx2 in the OM-EV group was also higher than that in the control group, among which the growth of DMP-1 was the most significant (see Fig. 4J and K). The mRNA expression of dentation-related genes was basically consistent with protein expression on days 7 and 14, except that the expression of DMP-1 was lower in the EV-treated groups compared to the control group on day 7 (see Fig. 4G and L).

### Pre-differentiation alters the microRNA profiles of SHEDs-derived EV

**Fig. 5:**
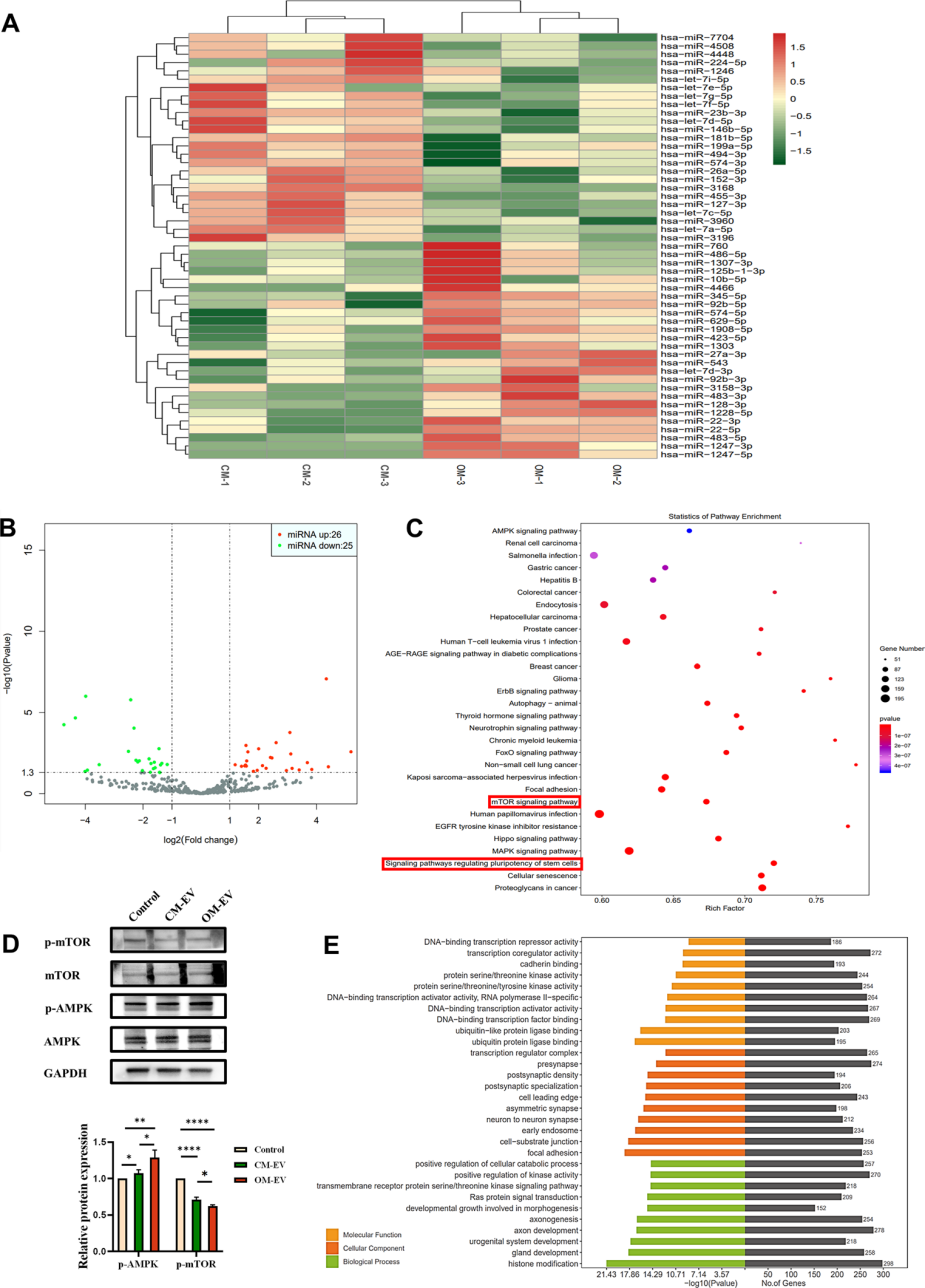
High-throughput sequencing for OM-EV and CM-EV. **A** Heat map of microRNA expression in OM-EV and CM-EV (*n* = 3). **B** Volcano plot showing significantly upregulated (red dots) and downregulated (green dots) microRNAs in OM-EV, compared with CM-EV. 51 microRNAs significantly changed, of which 26 microRNAs increased and 25 microRNAs decreased. **C** Kyoto Encyclopedia of Genes and Genomes (KEGG) pathway enrichment analysis. **D** Western blotting showing the expression of AMPK pathway-related proteins in DPSCs. **E** Gene Ontology (GO) analysis of the target mRNAs of the differentially expressed miRNAs (note: * *P* < 0.05, ** *P* < 0.01, and **** *P* < 0.0001)

MicroRNA sequencing revealed that pre-differentiation of SHEDs clearly alters microRNA profiles in OM-EVs. A total of 51 microRNAs were significantly altered in OM-EV, with 26 increasing trends and 25 decreasing trends (see Fig. 5A and B). The top 10 significantly upregulated microRNAs in OM-EV include hsa-miR-1247-3p, hsa-miR-1247-5p, hsa-miR-483-5p, hsa-miR-4466, hsa-miR-1228-5p, hsa-miR-1303, hsa-miR-760, hsa-miR-345-5p, and hsa-miR-483-3p. These miRNAs were considered to have a remarkable influence on the effects of OM-EV and were selected for further investigation.

Four databases, including TargetScan, miRDB, miRTarBase, and miRWalk, were employed to predict mRNA-miRNA interactions. To this end, the GO and KEGG analyses were performed. The performed Go analysis revealed that ubiquitin-protein ligase binding (GO:0031625), focal adhesion (GO:0005925), and histone modification (GO:0016570) were the most enriched GO functional terms, corresponding to molecular function, cell composition, and biological activity, respectively (Fig. 5D). Based on KEGG analysis, signaling pathways regulating pluripotency of stem cells, including TGF-β, MAPK, PI3K-Akt, Wnt, and AMPK, may be associated with the enhanced effects of OM-EV on the differentiation of DPSCs (see Fig. 5C). Furthermore, evidence of increased p-AMPK and decreased p-mTOR protein expression was found by Western blotting in OM-EV-treated DPSCs, suggesting that the AMPK-mTOR signaling pathway might be involved in the regulation of DPSCs function (see Fig. 5E).

### OM-EV-encapsulated hydrogel improved the survival and migration of DPSCs

**Fig. 6:**
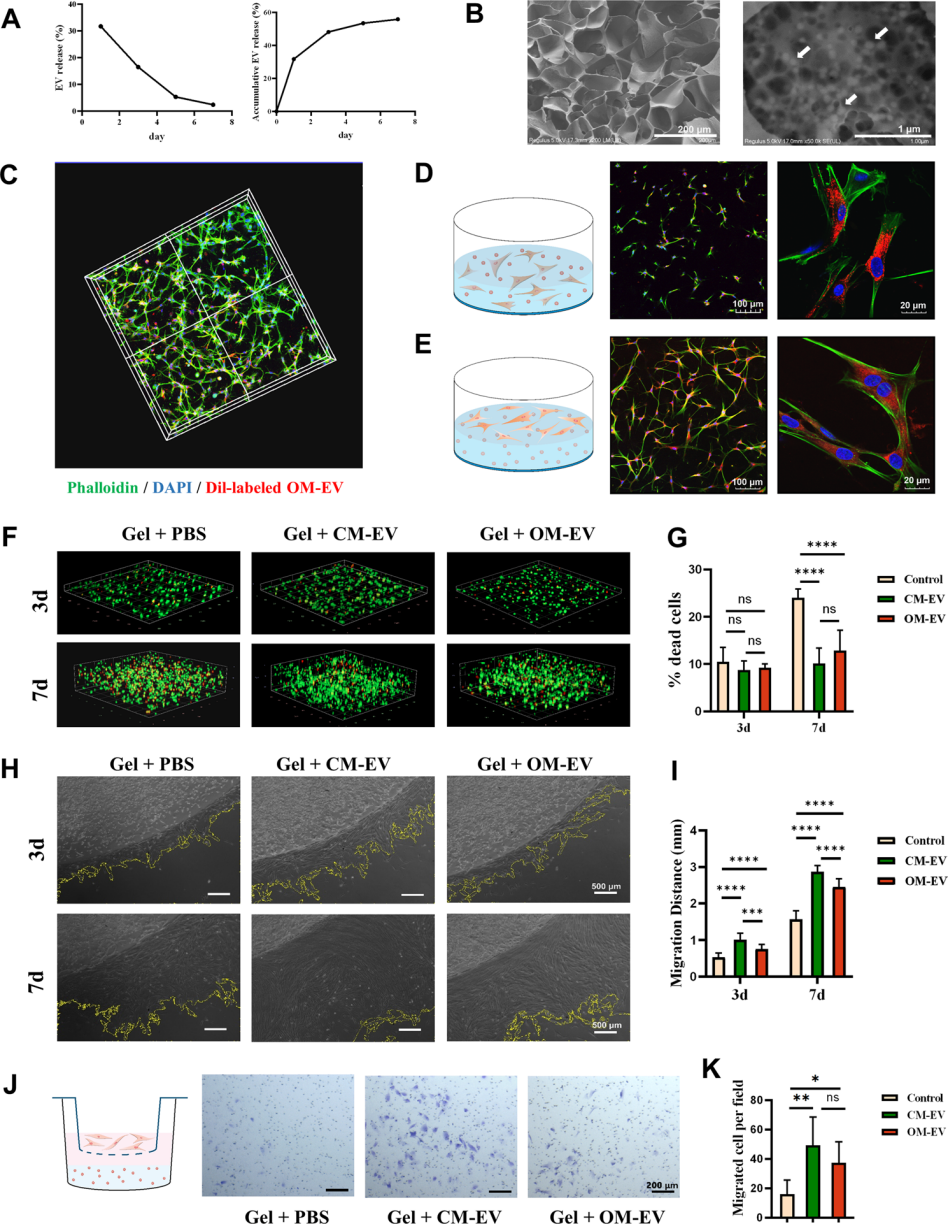
Characterization of OM-EV-encapsulated hydrogel. **A** Release curves of OM-EV obtained from the GelMA hydrogel. **B** Microstructure of OM-EV-encapsulated hydrogel characterized by SEM (note: white arrow shows the encapsulated OM-EV. The corresponding scale bars represent 200 μm and 1 μm, respectively). **C** Distribution of OM-EV and DPSCs in the OM-EV-encapsulated hydrogel demonstrated by 3D reconstruction with CLSM (note: OM-EV were labeled with Dil (red), while DPSCs and nuclei were stained with phallotoxins (green) and DAPI (blue), respectively). **D** Schematic representation and CLSM images illustrating the DPSCs embedded in the OM-EV-encapsulated hydrogel endocytosed OM-EVs released from the hydrogel (note: the corresponding scale bars are presented). **E** Schematic representation and CLSM images illustrating the DPSCs on the surface of the OM-EV-encapsulated hydrogel endocytosed OM-EVs released from the hydrogel (note: the corresponding scale bars are presented). **F** Live/Dead staining of DPSCs within the OM-EV-encapsulated hydrogel (note: live and dead cells have been labeled with green and red colors, respectively). **G** The proportion of dead cells within the OM-EV-encapsulated hydrogel. **H** The migration ability of DPSCs within the OM-EV-encapsulated hydrogel (note: the scale bar length is 500 μm). **I** Migration distance of DPSCs within the OM-EV-encapsulated hydrogel. **J** Schematic illustration and representative images showing the effect of OM-EV-encapsulated hydrogel on the migration ability of DPSCs tested by the transwell assay (note: Scale bar represents 200 μm). **K** Migrated cells per field in each group (note: Error bars represent the values of “mean ± s.d.”; * *P* < 0.05, ** *P* < 0.01, *** *P* < 0.001, and **** *P* < 0.0001)

Based on the results of the CCK-8 assay, EdU assay, and morphological observations, it was proved that a 5% concentration of GelMA has the best biocompatibility for constructing OM-EV encapsulated hydrogel (see Fig. 2S). The OM-EV release curve from the 5% GelMA hydrogel displayed that more than 30% of the loaded OM-EV was released on the first day, and half of the OM-EV was continuously released, reaching a plateau within 7 days at 37 °C in a humidified environment (Fig. 6A). Based on the release efficiency and the optimal concentration of OM-EV, a hydrogel complex composed of 500 µg/mL OM-EV and 5% GelMA was prepared. The representative SEM micrographs presented the binding of OM-EV to the hydrogel (Fig. 6B). We found that GelMA possessed a relatively typical leaf-like porous structure. OM-EV was revealed to adhere to the hydrogel and exhibit a regular round shape (white arrow).

CLSM showed the presence of Dil-labeled OM-EV (red) and phalloidin-labeled DPSCs (green), which were evenly distributed in the hydrogel when the DPSCs were co-cultured with the OM-EV-encapsulated hydrogel. The DPSCs were stretched in the hydrogel and were appropriately connected to each other (see Fig. 6C). The obtained results of the uptake assay indicated that the OM-EV released from the hydrogel could be efficiently endocytosed by the DPSCs embedded in the hydrogel and seeded on the hydrogel surface (see Fig. 6D and E).

The results of the live/dead assay revealed that most of the DPSCs embedded in the hydrogel maintained their viability. On day 3, the numbers of dead cells were comparable in the three groups (PBS, CM-EV, and OM-EV). As time progressed, the proportion of dead cells in the OM-EV group was lower than that of the control group on day 7, while there was no statistical difference compared with the CM-EV group (see Fig. 6F and G).

Accordingly, the migratory ability of hydrogel-embedded DPSCs seeded on the hydrogel surface was signally improved by treatment of the encapsulated hydrogel with OM-EV (see Fig. 6H–K).

### OM-EV-encapsulated hydrogel increased the formation of pulp-dentin complex in vivo

**Fig. 7:**
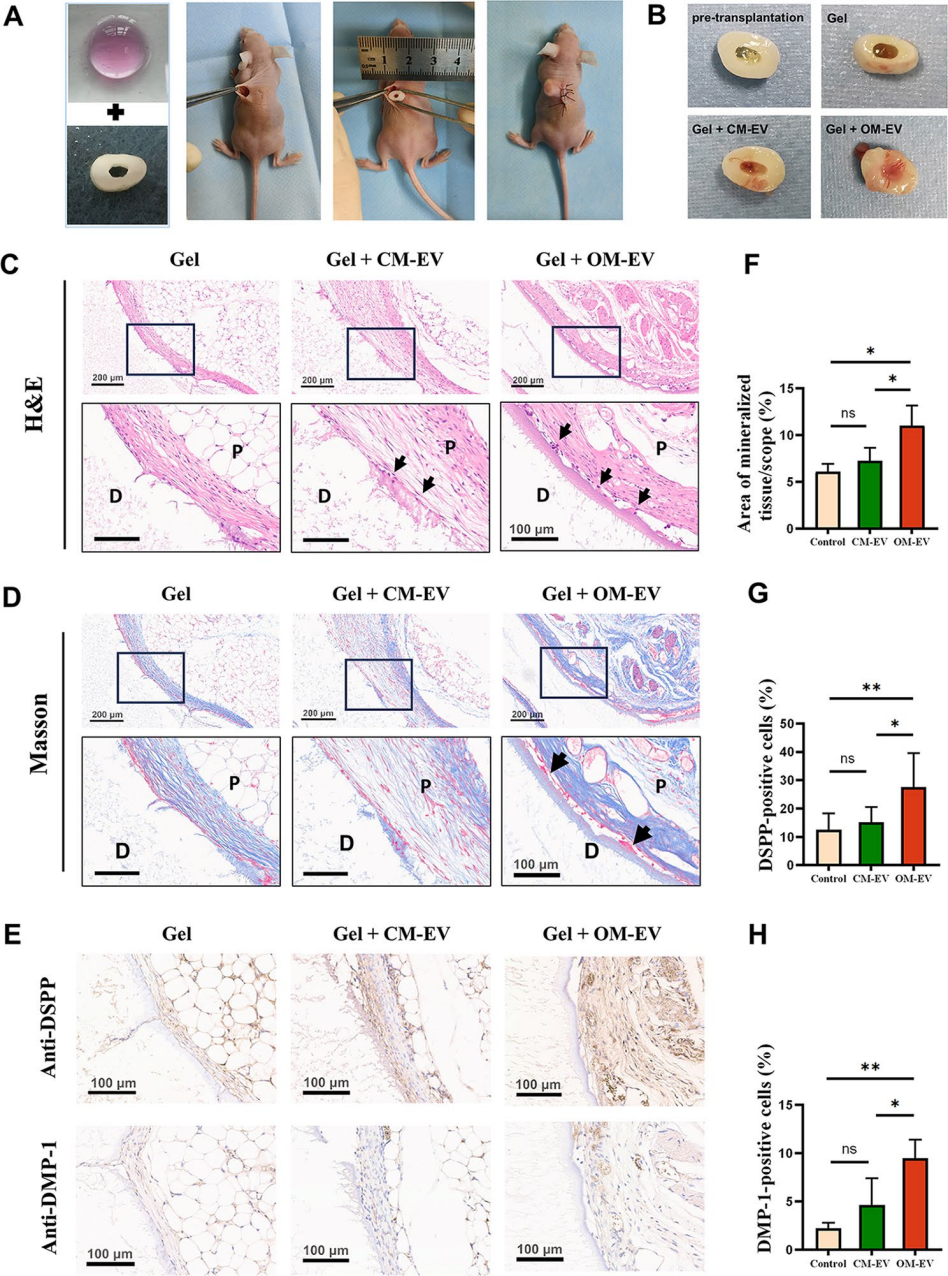
OM-EV-encapsulated hydrogel increased the formation of pulp-dentin complex in vivo. **A** Steps used for subcutaneous transplantation of tooth root slices in nude mice. **B** General view of the tooth root slices before transplantation and after 8 weeks of subcutaneous transplantation. **C** Representative images of H&E staining of sections from tooth root slice (note: P: dental pulp-like tissue, D: dentin, M: mineralized tissue (black arrow); Scale bar lengths are set as 200 and 100 μm). **D** Representative images of Masson staining of sections from tooth root slice (note: the scale bar lengths are 200 μm and 100 μm, respectively). **E** Representative images of immunohistochemical staining showing the upregulated expression of odontogenic markers (DSPP and DMP-1) (note: scale bar represents 100 μm). **F** Quantification analysis of mineralized tissue area fraction. **G** Quantitative analysis of DSPP-positive cells. **H** Quantitative analysis of DMP-1-positive cells (note: Error bars represent the values of “mean ± s.d.”; * *P* < 0.05, ** *P* < 0.01, *** *P* < 0.001, and **** *P* < 0.0001)

After 8 weeks, tooth root slices were harvested. The general view of tooth root slices in various groups revealed that more tissue formation occurred in the root canal in the Gel + OM-EV group, while transparent hydrogel can still be seen in the control group (see Fig. 7B). The histological analysis by H&E staining and Masson staining (see Fig. 7C and D) demonstrated that new mineralized tissue (black arrow) was formed in the OM-EV-encapsulated hydrogel group. The quantification of mineralized tissue indicated that more dentin-like tissue was formed in the root canal in GelMA hydrogel loaded with OM-EV compared to CM-EV (*P* < 0.001). The positive expression of odontoblastic markers (DSPP and DMP-1) was detected in IHC staining (see Fig. 7E and F).

**Scheme 1:**
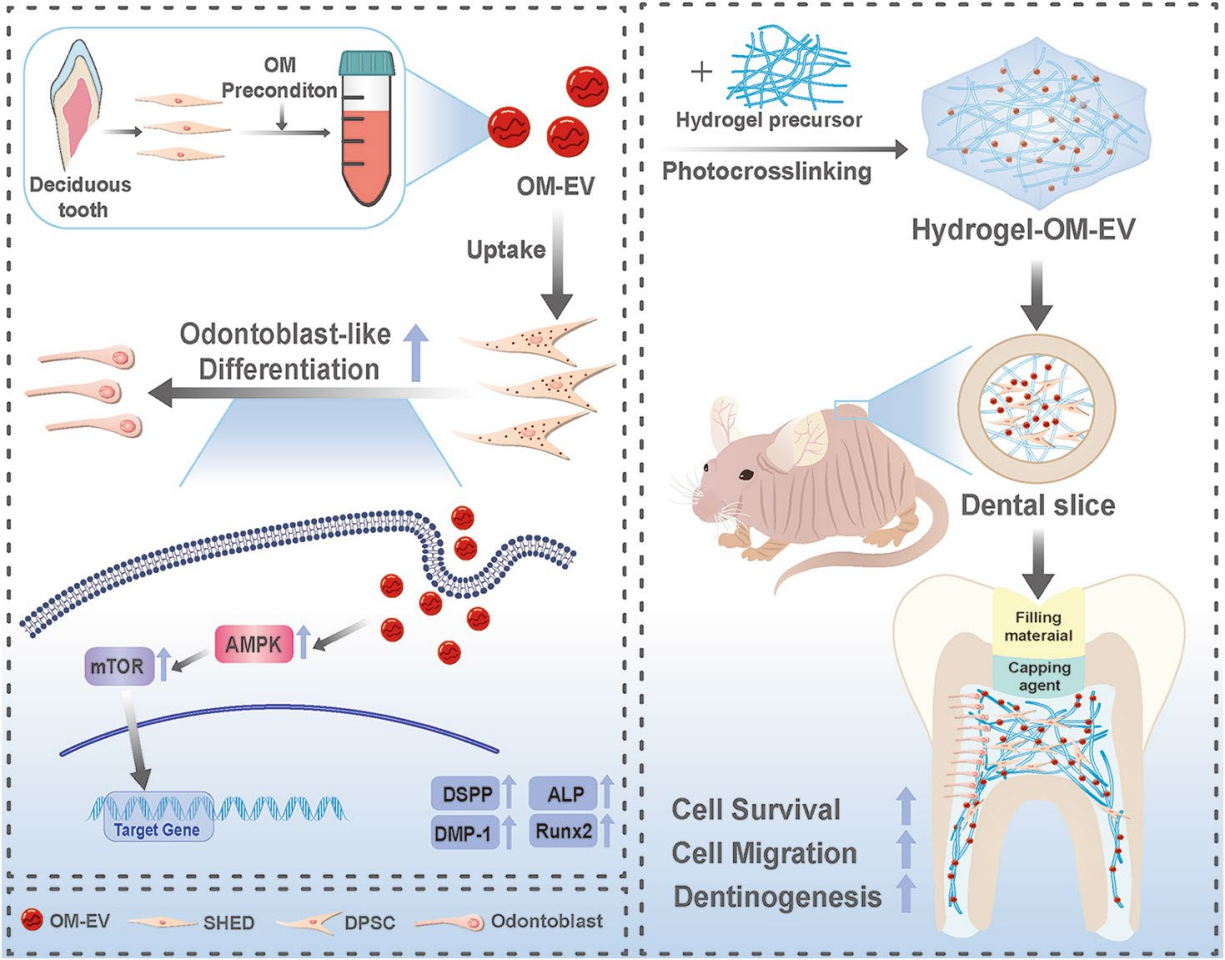
Graphical abstract of the present study and the potential application of OM-EV-encapsulated hydrogel for pulp regeneration

## Discussion

Pulp necrosis in young permanent teeth is able to cause many problems such as insufficient root length and thin root wall. Pulp regeneration using a combination of stem cells, scaffolds, and growth factors is a promising alternative to regenerate pulp tissue and promote dentinogenesis. Due to the higher potential of SHEDs differentiating into osteoblasts and endothelial-like cells, current bioengineering approaches have focused on utilizing SHEDs secreted by EVs for bone regeneration and pulp regeneration [27]. However, previous investigations on the ability of SHED-derived EVs to promote odontogenic differentiation are critically limited. In this study, we employed the odontogenic induction medium (OM) of SHEDs as a specific stimulus to generate functional EVs (OM-EVs) with a superior capacity to stimulate the odontogenic differentiation of DPSCs and combined the EVs with a photo-crosslinked hydrogel to prevent aggregation and the explosive release of EVs. We successfully determined that OM-EV leads to increasing the formation of mineralized nodules and the expression of dentinogenesis-related genes in DPSCs, while also having a mild promotional effect on cell proliferation and migration at a concentration of 20 µg/mL. Through detailed characterization and validation, the results are indicative of the fact that the OM-EV-encapsulated hydrogel is capable of releasing EVs sustainably in vitro with good biocompatibility and promoting mineralized tissue formation in a tooth root slice model which was subcutaneously transplanted in nude mice. This is the first to examine the role of SHEDs-derived OM-EV incorporated with GelMA hydrogel in dentinogenesis. Our findings provide insights into the potential application of OM-EV-encapsulated hydrogel for pulp regeneration.

We first extracted and identified functionalized OM-EV from culture supernatants of pre-differentiated SHEDs. According to the guidelines proposed by ISEV in 2018, the extracted EVs were characterized by NTA, TEM, AFM, and Western Blotting. Our results indicated that OM-EV had larger particle sizes, as well as higher particle and protein concentrations, compared to CM-EV. This fact suggests that after odontoblast-induced differentiation, the amount of EVs secreted by SHEDs was increased, and the cargo carried, such as proteins and miRNAs, may have changed. Furthermore, the higher secretion efficiency of OM-EV may be attributed to changes in environmental factors [28]. Concerning the particle size results obtained by TEM, AFM, and NTA, some discrepancies were obtained in the present investigation, which may be due to differences in detection environments. Unlike TEM and AFM, the NTA is able to reflect the dynamic diameter of the fluid to analyze the dynamic movement process of particles in solution. The obtained results revealed that larger particles will have a greater impact on detection results and may cause slightly larger particle sizes [29].

The effects of EVs on recipient cells generally involve two processes: one is the signal transmission mediated by endocytosis, and the other is the gene expression and physiological activities of recipient cells mediated by EV cargo [13]. Among them, the selective uptake of EVs by recipient cells is mainly regulated by three mechanisms: receptor-ligand interaction, direct membrane fusion, and receptor-cell phagocytosis [30]. At present, evidence on the endocytosis and metabolic duration of MSC-derived EVs is limited. In the present investigation, we observed the accumulation of OM-EVs in DPSCs for up to 7 days and confirmed the internalization of OM-EVs by DPSCs with CLSM. On the third day of co-culture, the Dil-labeled EVs were not only internalized into the cytoplasm but also attached to the cell membrane, indicating that these three pathways jointly regulate the uptake of EVs by DPSCs. The results also showed that OM-EV accumulation peaked on days 3–5 of co-culture. In addition, Dil-labeled OM-EV was still observed in the cytoplasm of DPSCs at day 7, indicating that OM-EV had a long residence time in DPSCs. This long retention time may result in a long-term impact of OM-EV on cells, inspiring us to investigate the effects of OM-EV on cell proliferation and odontogenic differentiation.

Although the impact of SHED-derived EVs on cell proliferation and migration has been extensively examined, it is still unclear whether EVs from pre-differentiated SHEDs retain their proliferative effects. Hu et al. [15] demonstrated that 30 µg/mL OM-Exo derived from DPSCs could remarkably promote the odontogenic differentiation of cells, but its effects on cell proliferation and migration are still unclear. In this study, we determine the minimum effective concentration of OM-EV to be 20 µg/mL (approximately 2.4 × 10^10^ EVs/mL) based on the CCK-8, EdU, and cell cycle analyses. Subjected to this concentration, the proliferation of DPSCs was slightly improved, and the migration ability of DPSCs was significantly enhanced. Wu et al. [5] elucidated that SHED-derived exosomes promoted the proliferation of human umbilical vein endothelial cells in a concentration-dependent manner within the range of 5–10 µg/mL. Zhuang et al. [31] showed that 0–50 µg/mL SCAP-Exo had no substantial effect on the proliferation of bone marrow mesenchymal stem cells, but 20 and 50 µg/mL SCAP-Exo obviously increased DSPP expression. The differences in concentration effects may arise from variations in the sources of EVs and their target cells. Nevertheless, we found that 20 µg/mL is about 20 times the amount secreted by the cells themselves based on the calculations of the present study, which may be a reference for further investigations. It was a pity that higher concentrations of EVs derived from SHED were not examined due to the low isolation efficiency of EVs. There only exist a few studies that have been devoted to the optimal concentration. Zhang et al. [32] found that EVs from Hertwig’s epithelial root sheath cells had the best influence in promoting the proliferation and migration of DPSCs at 80 µg/ mL in the range of 0–240 µg/mL. Guo et al. [33] examined the osteogenic effects of SHED-derived exosomes under various concentrations (i.e., 0, 30, 60, 90, 120, and 150 µg/mL) and reported that 90 µg/mL exosomes were able to noticeably promote Runx2 expression. Future investigations should focus on the optimal dosage of EVs and whether high concentrations of EVs have adverse effects on recipient cells and organs.

Through a series of experiments in vitro, it was demonstrated that both CM-EV and OM-EV were capable of enhancing the odontogenic potential of DPSCs at a concentration of 20 µg/mL, and the effect of OM-EV was better than that of CM-EV, which is consistent with previous investigations [13–15]. Alizarin red staining revealed that compared to CM-EV and the control group, OM-EV substantially incorporated into the formation growth of mineralized nodules, particularly on day 14. The ALP is commonly regarded as an early marker of odontogenic differentiation and is noticeably expressed after one week of induction [21, 34, 35]. Therefore, we performed ALP staining and ALP activity detection only on day 7. However, the discrepancy between OM-EV and CM-EV was not noticeable, indicating a similar trend in the qPCR and Western blot. Regarding the results of qPCR and WB, there are several interesting points worth discussing. The results revealed that OM-EV upregulated the expressions of odontogenic-related markers by approximately 1.1 to 1.8 times. OM-EV showed limited capacity in inducing DMP-1 expression on day 7, but increased the expression on day 14. Conversely, up-regulation of DSPP was more pronounced on day 7. The possible explanation is the diverging expression of multiple genes at different time during odontogenic differentiation. Similar phenomenon was observed in Sangsuwan’s study, in which DSPP upregulated at an early phase (day 1–7) while DMP-1 upregulated on day 28–42 [36]. Since ALP and Runx2 are usually highly expressed in the early stages of differentiation [37], it is reasonable that they were slightly increased in the OM-EV group after 14-day OM-EV induction. Furthermore, we hypothesized that 14-day OM-EV induction may involve a negative feedback mechanism leading to negligible expression of ALP, Runx2, and DSPP. However, the exact mechanism has not yet been confirmed. Since relatively low concentrations and duration of induction were employed in the experiments, higher concentrations of OM-EV and longer incubation time are highly required in future studies to obtain more convincing results.

It is well known that EVs carry RNA and proteins to participate in intercellular communications. Since miRNAs are regarded as one of the vital “cargos” carried by EVs and act as important post-transcriptional gene regulatory factors [38], herein, we apply high-throughput sequencing to detect the miRNAs profiles in OM-EV. We found 51 differentially expressed miRNAs through microRNA sequencing. Among the upregulated 26 miRNAs, has-miR-483-3p [39–41], has-miR-486-5p [42], has-miR-22-3p [43], and has-miR-27a-3p [44, 45] have been confirmed to promote osteogenesis; however, their effects on dentinogenesis remain to be examined. A series of studies have revealed that the promotion of odontoblast differentiation by EVs from dental mesenchymal stem cells may be achieved through regulating multiple signaling pathways (such as Wnt/β-catenin [32], p38/MAPK [13], and TGF-β/smads [15]) by microRNA, and proteins were carried by EVs. In a previous article by our team, we also suggested the correlation between the AMPK/mTOR pathway and the odontogenic differentiation of DPSCs [21]. The obtained results of pathway analysis and Western blotting revealed that the activation of the AMPK/mTOR pathway may play a pivotal role in regulating the odontogenic differentiation of DPSCs by OM-EV. AMPK is a serine/threonine protein kinase conserved throughout evolution, acting as an energy sensor and regulating cell metabolism to maintain energy homeostasis [46]. As a major regulator of growth, mTOR is capable of sensing and binding to various nutritional and environmental factors, including growth factors, energy levels, cellular stress, and amino acids, promoting ribosome biogenesis and synthesis of proteins, nucleotides, fatty acids, and lipids, while inhibiting catabolic processes such as autophagy [47]. Therefore, we hypothesize that by delivering specific miRNA to recipient cells, OM-EV may activate AMPK and downregulate mTOR, thus altering the metabolic state of the cell and leading to the upregulation of the expressions of DSPP, DMP1, and Runx2, and the odontogenic differentiation of DPSCs.

Based on sufficient pieces of evidence that OM-EVs promote the dentinogenic differentiation of DPSCs, we developed an injectable hydrogel encapsulated with OM-EV for in vivo testing. The exploitation of EV in solution may lead to aggregate and explosive release. The hydrogel scaffolds should be properly introduced to provide a hydrophilic 3D polymer network for EV protection and sustained release [48]. In a previous article on the role of preconditioning of EVs in promoting cell differentiation, the combination of EVs and scaffold was not revealed and it was unclear whether EVs embedded in a hydrogel might have the same effects as free EVs [15]. It has been proven that EVs encapsulated in GelMA hydrogels are able to maintain their particle number, size, structure, and protein [48]. For this reason, we chose GelMA as the scaffold for EV encapsulation in the present study. We determined the optimal concentration of GelMA through CCK-8 assay, Edu assay, and cell morphology observation. According to the GelMA instructions, when cured under 405 nm light for 30 s, the compressive modulus of GelMA reached 3.34 kPa, which is comparable to a dental pulp [49]. In the constructed OM-EV-encapsulated hydrogel, the three-dimensional structure of the hydrogel suitable for cell growth and differentiation was revealed by SEM. The GelMA hydrogel possessed a loose porous structure, and thereby, it is capable of maintaining the biological activity of EVs and DPSCs. The embedded OM-EV could be stably released and effectively internalized by DPSCs inside and outside the system, suggesting that OM-EV within the hydrogel complex is able to enter DPSCs to exert their biological function.

In addition to providing mechanical support, scaffolds, when combined with EVs, can also provide a three-dimensional (3D) space for controlled EV release [20, 50]. This allows EVs to fully exert their therapeutic effects [51, 52], making the combination of scaffolds and EVs a promising approach in regenerative therapy. Different from traditional inorganic scaffolds, EVs in this study were able to be continuously released from the hydrogel for 7 days, which enabled EVs to have a long-term effect in an in vivo model. As expected, in the in vivo study, after 8 weeks of subcutaneous transplantation, more mineralized tissue was observed in the Gel + OM-EV group with a higher level of DPSS and DMP-1 expression, indicating that the hydrogel encapsulated with OM-EV exhibits superior potential to promote dentinogenesis.

It is emphasized here that the limitations of the present investigation are undeniable. In the present research work, the release of OM-EV from the hydrogel complex was more than 30% on the first day, which was similar to that in other hydrogel scaffolds [53]. In other words, on the first day, the concentration of OM-EV loaded onto the targeted cells was up to 150 µg/mL. It was a pity that the in vitro effects of high-dose OM-EV on the proliferation and migration of DPSCs were not revealed in the present study. However, DPSCs co-cultured with the OM-EV hydrogel system exhibited superior cell viability and migration to the control group without OM-EV. Such a fact proves that the concentration of OM-EV released from the hydrogel complex incorporates into the enhancement of the proliferation and migration of DPSCs. We will further explore the relationship between high concentrations of EVs and their effects on cell proliferation as well as migration in future investigations. Furthermore, this study focused on examining the role of OM-EV-encapsulated hydrogel in promoting dental pulp regeneration; however, the mechanism of OM-EV causing dentinogenesis was not fully investigated. In the near future, we will conduct further studies to elucidate the specific mechanisms by which OM-EV-carried miRNA affects the odontogenic differentiation of DPSCs through the AMPK/mTOR pathway.

## Conclusions

In summary, we successfully extracted functional EVs derived from preconditioned SHEDs to upregulate the odontogenic differentiation of DPSCs. An injectable hydrogel complex containing OM-EV and GelMA was also constructed and characterized, leading to promoting proliferation, and migration of DPSCs in vitro, and facilitating dentinogenesis in vivo. To the best of our knowledge, this is the first investigation to combine SHED-engineered EV with hydrogel for pulp regeneration. Our findings strongly reveal the effect of OM-EV-encapsulated hydrogel in dentinogenesis and provide a promising biomimetic tool for pulp regeneration.

### Electronic supplementary material

Below is the link to the electronic supplementary material.

Supplementary Material 1

Supplementary Material 2

Supplementary Material 3

## Data Availability

The datasets used and/or analyzed during the current study are available from the corresponding author upon reasonable request.

## Abbreviations

SHEDs: Stem cells from human-exfoliated deciduous teeth
DPSCs: Dental pulp stem cells
MSCs: Mesenchymal stem cells
OM: Odontogenic induction medium
OM: EV-EV derived from OM
CM: Conventional medium
CM: EV-EV derived from CM
TEM: Transmission electron microscope
AFM: Atomic force microscopy
NTA: Nanoparticle tracking analysis
CLSM: Confocal Laser Scanning Microscopy
SEM: Scanning emission microscope
CCK: 8-Cell Counting Kit-8
DSPP: Dentin siolophosphoprotein
DMP: 1-Dentin matrix protein 1
ALP: Alkaline phosphatase
Runx2: Runt-related transcription factor 2
H&E: Hematoxylin and eosin
GO: Gene Ontology
KEGG: Kyoto Encyclopedia of Genes and Genomes
