# Supplementary figures and images for: Functional extracellular vesicles from SHEDs combined with gelatin methacryloyl promote the odontogenic differentiation of DPSCs for pulp regeneration
Source: J Nanobiotechnology. 2024 May 17;22:265. doi: 10.1186/s12951-024-02542-0 (PMC11102175; doi:10.1186/s12951-024-02542-0)

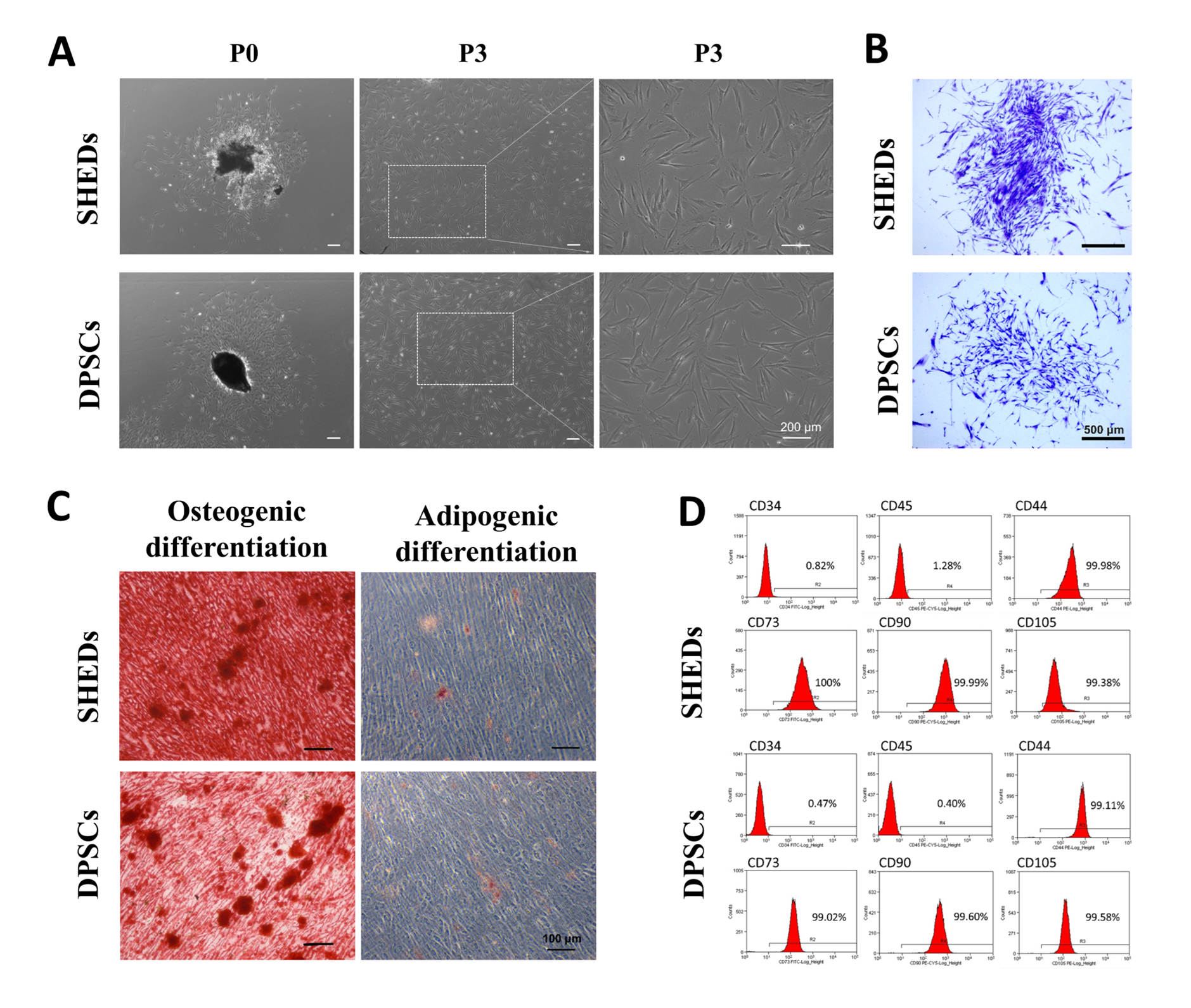

Supplement: Supplementary file 1 — Supplementary Material 1 [file 12951_2024_2542_MOESM1_ESM.tif]

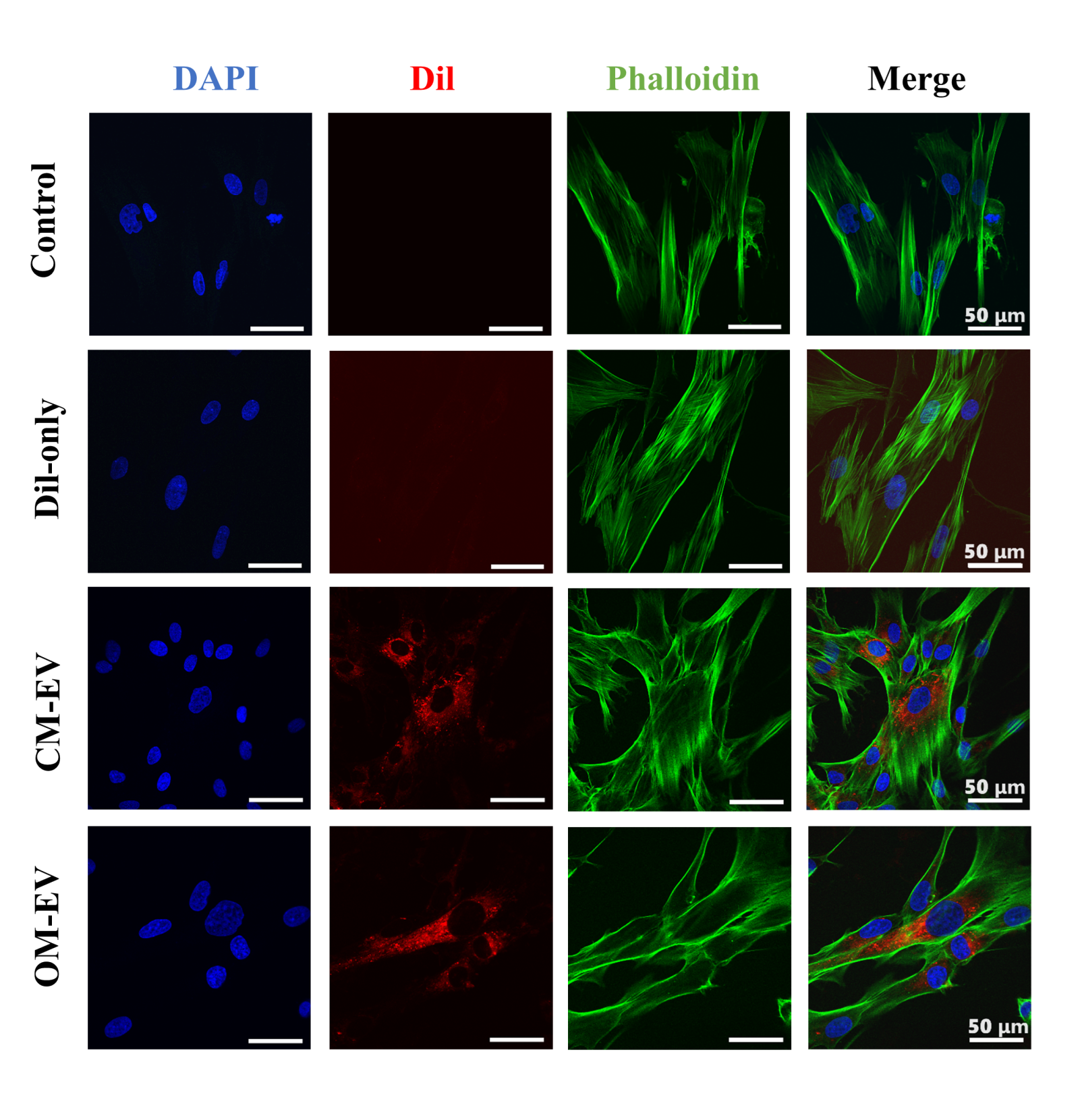

Supplement: Supplementary file 2 — Supplementary Material 2 [file 12951_2024_2542_MOESM2_ESM.tif]

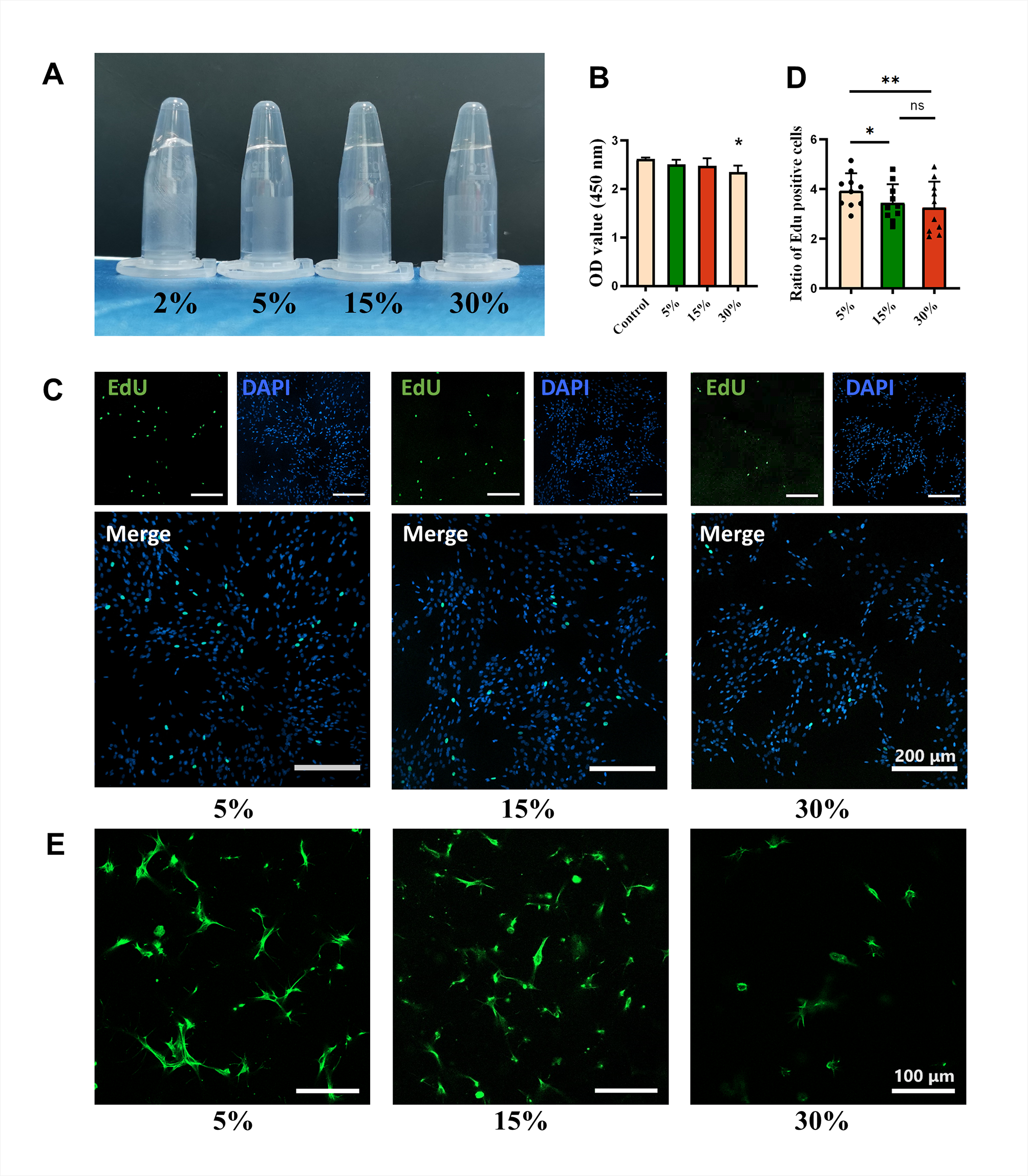

Supplement: Supplementary file 3 — Supplementary Material 3 [file 12951_2024_2542_MOESM3_ESM.tif]
